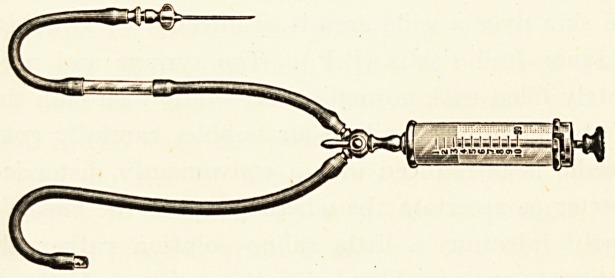# The Intravenous Administration of Neo-Salvarsan in Syphilis

**Published:** 1913-03

**Authors:** J. A. Nixon

**Affiliations:** Physician and Physician-in-Charge of Skin Department, Bristol Royal Infirmary.


### THE INTRAVENOUS ADMINISTRATION OF NEO-SALVARSAN IN SYPHILIS.

BY

J. A. Nixon, M.B. Cantab., F.R.C.P.

*Physician and Physician-in-Charge of Skin Department, Bristol Royal Infirmary.*

Neo-Salvarsan is a new derivative of Salvarsan, obtained by Professor Ehrlich by the action of Formaldehyde-Sulphoxylate upon Salvarsan (Dioxydiamidoarsenobenzol). The resultant compound contains as its active constituent Dioxydiamido-arsenobenzol-monomethane-sulphinate of sodium and bears the number "914" in Ehrlich's series.

The first great advantage of Neo-Salvarsan over Salvarsan is the ease with which it dissolves in water. Unfortunately, like many other organic arsenical compounds, it is unstable, and readily oxidises, giving rise to products which are exceedingly toxic.

My experience with this drug is short, but I am convinced that it possesses many advantages over the original Salvarsan in the treatment of syphilis. The technique of administration is comparatively simple, but it demands close attention to detail.

Neo-Salvarsan may be given intra-muscularly or intravenously; but the latter is the method with which I propose to deal.

McDonagh's method of using a syringe for intravenous administration is excellent, and with slight modifications Messrs. Ferris & Co. have made for me a compact injection apparatus, which fits on to an ordinary 20 c.c. Ehrlich-Hata "Record" Syringe. To the nozzle of the syringe a three-way stop-cock is screwed on, the needle of the syringe is connected with one branch of the stop-cock by a piece of rubber tubing about eight inches long, into which a "window" of glass tube is inserted two inches from the needle. To the third branch of the stop-cock a piece of rubber tubing twelve inches long is attached, through which the syringe can be filled from the solution of Neo-Salvarsan. A turn of the tap enables the contents of the syringe to be injected through the needle into the vein.

The preparation of the solution of Neo-Salvarsan is carried out as follows. Into a sterilised beaker is poured, with aseptic precautions, a sufficient quantity of the twice-distilled sterilised water to allow 25 c.c. of water for every 0.1 gramme of Neo-Salvarsan. The twice-distilled water is absolutely essential to avoid a febrile reaction after the injection ; and it is advisable to sterilise in an autoclave the water in a flask after re-distillation. The re-distillation and autoclaving should be done not longer than the day before injection. The phial of Neo-Salvarsan is then opened. The neck of the phial is rendered aseptic by wiping with a cotton-wool swab soaked in absolute alcohol, and filed off with a small file sterilised in the flame of a spirit lamp. Heat must not be applied to the phial itself. The contents of the phial, consisting of a finely-divided yellow powder, is shaken on to the surface of the water, and allowed to dissolve spontaneously without shaking or stirring. If the solution is not perfectly clear at the end of ten minutes, it should not be stirred or filtered ; more distilled water should be added. If the water was perfectly clear of all solid particles, there will be no need of filtering : Neo-Salvarsan will dissolve completely, provided sufficient water is added. A turbid solution indicates an insufficiency of water.

A second beaker should be prepared, containing sterilised normal saline solution, made from twice-distilled water. A vein at the bend of the elbow is meanwhile made to distend by placing a rubber tourniquet, secured by artery forceps, round the upper arm, taking care that the radial pulse is not obliterated.

The skin over a wide area is disinfected by swabbing once with Liquor Iodi Fortis (B.P.). The syringe and needle are completely filled with normal saline solution through the spare end of rubber-tubing, and all air bubbles carefully got rid of. The needle is introduced into a conveniently distended vein. It is better to ascertain the whereabouts of the needle's point by gently injecting a little saline solution rather than by attempting to suck up blood into the syringe. If the point is not in the vein, subcutaneous infiltration will immediately be visible as a slight swelling. Provided no such swelling appears, a few c.c. of saline solution should be injected into the vein, and the plunger withdrawn until pure blood is seen in the glass window-tube. This must be immediately re-injected, or clotting may occur in the needle.

Having made sure that the needle is properly introduced into the lumen of the vein, the needle can be kept in position by tying a piece of gauze round the tubing just above the junction with the needle (if gauze is used instead of tape or a bandage a single knot suffices, and will not slip). The tourniquet is then relaxed : a most important preliminary, and one sometimes forgotten. By a turn of the stop-cock the saline solution is emptied out of the syringe, the spare end of tubing placed in the Neo-Salvarsan, and the syringe filled with Neo-

Salvarsan solution. The stop-cock is turned over to the needle, and the solution injected into the vein. This alternate filling with the Neo-Salvarsan and injection into the vein is continued by appropriate reversing of the stop-cock until the whole of the solution is injected. A final syringeful of saline solution should be injected to wash through the whole of the Neo-Salvarsan into the vein, and avoid the possibility of infiltration of the subcutaneous tissues with Neo-Salvarsan on withdrawing the needle.

If the subcutaneous swelling appears during injection, either the needle has become dislodged or the vein has been transfixed. A little saline solution should be injected to dilute the Neo-Salvarsan in the tissues, and a fresh vein must be taken for the remainder of the injection. The pain of subcutaneous injection of Neo-Salvarsan is intense and must be avoided.

At the end of the injection a pad of sterilised gauze is bandaged over the spot, as bleeding sometimes take place. Carried out in this way, the operation should be followed by no reaction and no rise of temperature. Patients prefer the actual injection to be performed as quickly as possible. Using 0.9 grammes of Neo-Salvarsan in 250 c.c. of water, I find the injection may be made in just over five minutes. The solution is injected at room-temperature about 55°–60° F. On a very cold day the flask of water may be warmed by standing in hot water prior to making the solution ; but the solution must on no account be warmed after the Neo-Salvarsan has been added. The solution must be freshly prepared at the time of injection, and it must not be carried about or agitated. With these precautions the danger of toxic decomposition products being formed from the Neo-Salvarsan is averted.

Every instrument, towel, swab, etc., to be used at the operation is sterilised by boiling or super-heated steam. Sterilised towels are clipped round the patient's arm, leaving only the iodine-painted area exposed. Sterilised towels are placed under the arm and over a sufficient surface of the table or bed on which the arm rests to ensure that the syringe and its various attachments may be safely laid down if necessary upon a sterilised surface.

I find it convenient to stand the two beakers of Neo-Salvarsan and saline solution in a double pickle jar stand, so that both may be carried about and handed to me simultaneously. Solid particles or air-bubbles might prove fatal if injected into the vein. Should an air bubble by chance appear at the glass window tube, it must be at once drawn back again into the syringe and allowed to float up to the top. The injection may then be continued. An air bubble of considerable size in the syringe does no harm if the syringe is held perpendicularly, and the plunger is not pushed home at each thrust so as to empty the syringe. Personally, I prefer to keep a fairly large air bubble at the top of the syringe, as small bubbles if they occur in the solution can be more easily dislodged from the sides of the syringe and made to incorporate themselves in the larger bubble, which I watch constantly.

The patients do not, as a rule, experience any unpleasant effects either during or after the injection. Some complain of a slight metallic taste at the end of the injection, but it is quite transitory. Occasionally a slight headache comes on two or three hours afterwards, and I find a cup of tea is the best remedy.

It is advisable to prepare the patients as if for an anæsthetic by administering an aperient on the previous night, and giving a very light breakfast on the morning of the injection. Ordinary meals may be taken after the injection. The patients should remain at rest, preferably in bed, for two hours ; if at the end of that time no unpleasant symptoms have occurred, I allow them to get up, provided they remain quiet. The first injection requires greater caution than subsequent ones so far as after treatment is concerned. If the patients stand the first injection well I allow them to get up and go out and about within a few hours after subsequent injections.

The dosage I employ is 0.75 gramme (No. V) for a first injection in a healthy man, with subsequent injections of 0.9 gramme (No. VI), at intervals of three days (in practice two injections per week), until four or five injections have been given. The Wassermann reaction in an early case of syphilis is usually positive up to four injections; if it is negative a week after the fifth injection I do not repeat the injection unless a later test shows a positive reaction again. If after the fifth injection the reaction is not negative, I give a sixth or seventh injection, with intervals of a week, taking a Wassermann test a week after each injection, and being guided as to a repetition of the dose by its results. I discontinue mercury for at least a fortnight before the first Wassermann test is made, but afterwards combine the treatments. Before finally deciding that no further Neo-Salvarsan is necessary I make a final Wassermann test, preceded by a fortnight without mercury.

Neo-Salvarsan is apparently contra-indicated in alcoholic debilitated subjects, or in those suffering from cirrhosis of the liver. I make it a rule to examine the urine beforehand, ascertaining with particular care that the quantity secreted is normal, that the specific gravity is within healthy limit, and above all that the excretion of urea is sufficient.

A trace of albumen may depend upon the syphilitic infection, but the presence of casts or sugar should in the present state of our knowledge deter us from administering any of the arsenic compounds intravenously. In cases showing signs of any syphilitic involvement of the central nervous system, especially meningitis, spinal symptoms, psychoses, or optic neuritis, great caution should be exercised, and if Neo-Salvarsan is administered at all, the dosage should be very small at the outset.

Observing these precautions, Neo-Salvarsan offers a very valuable adjunct to the mercurial treatment of syphilis. The patient's infectivity is quickly minimised if not abolished, the visible signs of syphilis are speedily dispersed, *e.g.* the chancre, roseola and condylomata may disappear after the second injection. The symptoms are alleviated, and the Wassermann reaction changed from positive to negative, whatever this may really mean.

Time alone can demonstrate whether more solid advantages accrue, but alleviation to this extent is a gain not lightly to be disregarded. At least, Neo-Salvarsan offers the advantages aforesaid, as well as marked benefits in later manifestations, particularly tertiary lesions of the skin and mucous membranes. Some cases of locomotor ataxy and late nervous disorders have been improved.

In addition to these considerations it may be observed that Neo-Salvarsan if cautiously prepared and administered shows little or no toxicity, that it is easily dissolved, requires no elaborate apparatus and is injected at room-temperature. While the use of re-distilled water has proved the " reaction " after intravenous injections to depend more upon some ill-understood quality of the water than upon the drug employed.

REFERENCE.

*Salvarsan in Syphilis and Allied Diseases*, J. E. R. McDonagh. 1912.

## Figures and Tables

**Figure f1:**